# 

**Published:** 1899-02

**Authors:** 


### CONTENTS.

ORIGINAL ARTICLES: PAGE
- Digitalis. By PROF. H. D. HANSON, D.V.S. . . . . 67
- Advantages of Active Association Membership. By DR. S. STEWART . . . 71
- Municipal *versus* State Control of Tuberculosis. By EDWIN B. ACKERMAN, D.V.S. 75
- The Experiment Station Veterinarian as a Member of the State Board of Health. By M. H. REYNOLDS, D.V.M. . . . . 81
- Ovariotomy on the Bitch. By JOHN CORRIGAN . . . . 85
- Hypodermic Cathartics. By E. STANTON MUIR, PH.G., V.M.D. . . . . 87
- Veterinary Ethics. By JOSEPH W. PARKER . . . . 91
- Glanders and Its Suppression. By J. M. WRIGHT, M.D.C. . . . . 96
- Œsophagotomy. By W. H. RIDGE, V.M.D. . . . . 99

REPORTS OF CASES . . . . 102

THERAPEUTIC NOTES . . . . 104

DEPARTMENT OF SURGERY . . . . 107

CORRESPONDENCE . . . . 115

EDITORIALS . . . . 118

NECROLOGY . . . . 119

MARRIAGES . . . . 120

NEW PUBLICATIONS . . . . 121

SOCIETY PROCEEDINGS . . . . 123

